# Improved visible photocatalytic CO oxidation of CoTiO_3_ nanotubes by surface engineering and Au nanoparticle loading

**DOI:** 10.3389/fchem.2026.1884025

**Published:** 2026-08-03

**Authors:** Zhanyu Chu, Tianwei Dou, Zhuo Li, Lei Sun, Liqiang Jing

**Affiliations:** Key Laboratory of Functional Inorganic Materials Chemistry (Ministry of Education), School of Chemistry and Materials Science, International Joint Research Center for Catalytic Technology, Heilongjiang University, Harbin, China

**Keywords:** Au loading, CoTiO_3_ nanotube, electrospinning synthesis, oxygen vacancy, photocatalytic CO oxidation

## Abstract

The tubular structure photocatalyst, known for its excellent light absorption and mass diffusion properties, represents an attractive class of photocatalytic materials for the removal of toxic gaseous pollutants. However, the availability of adsorption activation sites and the separation of photogenerated charges remain insufficient, making improvements in these aspects meaningful. In this study, CoTiO_3_ nanotubes (denoted as CTO) are fabricated by a coaxial electrospinning technique followed by molten salt treatment. Subsequently, oxygen vacancies (Ovs) are created within the CTO by a vacuum-assisted hydrogen reduction strategy using NaBH_4_. Meanwhile, ultrafine Au nanoparticles (NPs) are uniformly anchored onto the surface of CTOvs by an *in situ* frozen photodeposition strategy, producing Au-CTOvs. The optimized Au-CTOv photocatalyst exhibits 2.7-fold enhancement in CO photooxidation compared to pristine CTO and is superior to N-doped TiO_2_. The experimental results confirm that the exceptional photoactivity can be attributed to three synergistic factors: the construction of the void and tubular structure accelerates mass transfer, the creation of Ovs on the nanotubes facilitates CO adsorption, and the anchoring of Au NPs improves charge separation and O_2_ activation.

## 1 Introduction

As a hazardous atmospheric contaminant, carbon monoxide (CO) is widely emitted from motor vehicle exhaust, industrial flue gas, incomplete fuel combustion, and other sources. It poses a significant risk to human health in terms of both acute and chronic toxicity, especially in confined environments, yet it often remains overlooked due to its colorless and odorless nature (Mahajan and Jagtap, 2020; Rose et al., 2017). Moreover, the absence of functional groups renders traditional filtration or adsorption technologies ineffective for satisfactory treatment, while thermal catalysis suffers from high energy consumption and is unsuitable for low-concentration CO removal (Li et al., 2020; Ma et al., 2023). Consequently, there is a growing demand for environmentally friendly and safe technologies capable of room-temperature oxidative removal of CO. Photocatalytic oxidation of CO with O_2_ to CO_2_ is considered a promising approach due to its ability to operate under mild conditions without excessive energy input (Eid et al., 2023; Ovcharov and Granchak, 2019; Wu et al., 2021). Within this technological landscape, TiO_2_ has attracted substantial attention as a benchmark photocatalyst (Rengifo-Herrera and Pulgarin, 2023; Ruan et al., 2023; Shoneye et al., 2021). However, the wide bandgap of TiO_2_ also limits its light absorption capability and photocatalytic activity. Compared with TiO_2_, narrow-bandgap semiconductors have a broader visible-light response and thus may exhibit higher photocatalytic activity. As a typical narrow-bandgap semiconductor, CoTiO_3_ exhibits strong light absorption within the 500–650 nm range, which can significantly enhance light utilization efficiency (Yang et al., 2024; Zhang H. et al., 2025). Moreover, CoTiO_3_ possesses a unique A_2_O_3_ structure, which makes it more suitable for surface engineering modifications (Wei et al., 2025). However, conventional CoTiO_3_ structures (such as nanoparticles (NPs)) often suffer from agglomeration, inefficient photogenerated charge separation, and O_2_ activation, compromising their photocatalytic activity. Structural regulation of CoTiO_3_ based on the electrospinning technique represents an effective and promising strategy for boosting its photocatalytic activity.

Our previous studies demonstrated the successful fabrication of inorganic oxide mesoporous hollow nanofibers using coaxial electrospinning combined with polyetherimide regulation (Dou et al., 2024; Dou et al., 2025). The creation of such a mesoporous hollow structure facilitates better mass transport, leading to improved photocatalytic performance. However, the formation of pure CoTiO_3_ requires heating to 650 °C, which causes the growth of NPs and the collapse of the porous structure, thereby degrading the photocatalytic activity. As a well-developed and attractive technique, the molten salt method has been proven to generate voids by fusing adjacent NPs, thus improving mass transfer and subsequent photocatalytic activity (Ding et al., 2025; Song et al., 2021). In previous studies, CoTiO_3_ was synthesized in molten alkali chlorides, yielding a material with a promising structure and properties (Wang et al., 2016). Therefore, incorporating an alkali chloride molten salt system is expected to create void structures within the nanotubes and enhance mass transfer.

Effective photogenerated charge separation and CO adsorption are key to improving photocatalytic CO oxidation performance. The introduction of oxygen vacancies (Ovs) on catalysts has been shown to promote the photogenerated electron transfer and the chemical adsorption of CO (Chen et al., 2024; Liu et al., 2022). Notably, the controllable creation of surface Ovs without generating Ti^3+^ (i.e., avoiding deep-level defects) in titanate is crucial but challenging. Although hydrogenation treatment with H_2_ is widely adopted, it often results in uncontrollable bulk defects during the high-temperature reduction process of titanate (Li et al., 2019). Creating a mildly reductive environment without external H_2_ gas is therefore an attractive strategy for constructing such desirable Ovs. The thermal decomposition of NaBH_4_ can release H_2_ gradually and, more importantly, a vacuum environment is more favorable for generating a localized reductive atmosphere at low temperature. Given that the bond energy of Co-O is lower than that of Ti-O in CoTiO_3_ lattice, such a mild reduction process preferentially forms surface Ovs while avoiding Ti^3+^ generation (Xu et al., 2020).

The electrons involved in the reduction reaction with O_2_ as the oxidant constitute another crucial aspect of CO oxidation (Zhang et al., 2021; Zhang et al., 2022). Cocatalyst engineering is a feasible approach to lowering the energy barriers for O_2_ activation and promoting charge separation (Gu et al., 2025; Liu et al., 2021). The activation of O_2_ by photogenerated electrons and the *in situ* generation of ⋅O_2_^−^ active species have been confirmed to enable CO oxidation. Efficient O_2_ activation is closely related to the electron-induced reduction reaction, and it can extract electrons from the 1D CoTiO_3_ nanotube efficiently. Given that Au with high electronegativity and a well-matched work function typically serves as a benign electron acceptor, anchoring Au NPs is expected to facilitate charge separation and O_2_ activation (He W et al., 2024). Hence, selecting an appropriate strategy to anchor Au NPs onto CoTiO_3_ nanotubes is key to improving CO oxidation, yet it remains a great challenge. Photoreduction is a feasible approach for anchoring Au onto supports, but controlling the particle size during deposition is difficult. The freezing-assisted approach has been shown to prevent aggregation of metal atoms during synthesis (Zhao et al., 2024). In addition, under photoexcitation, partial electrons accumulate surrounding Ov sites over CoTiO_3_, and such charge enrichment centers are favorable for attracting Au^+^. Hence, we speculate that Au ions can be partially reduced and positioned around oxygen vacancies to form anchored Au NPs on CoTiO_3_ via *in situ* photoreduction assisted by freezing treatments.

This study demonstrates the successful preparation of ultrafine Au NP-loaded CoTiO_3_ nanotubes with Ovs (Au-CTOv)s through a multi-step process for photocatalytic CO oxidation. Initially, the CoTiO_3_ nanotubes are controllably fabricated using the coaxial electrospinning technique and molten salt method. Subsequently, Ovs are created within the CTO by a vacuum-assisted hydrogen reduction strategy using NaBH_4_. Meanwhile, ultrafine Au NPs are primarily anchored onto the surface of CTOvs by a light-induced *in situ* deposition strategy under a frozen environment created by liquid N_2_. Experimental results reveal that the enhanced photoactivity is mainly attributed to (1) the construction of the voids and tubular structure, which accelerates mass transfer; (2) the creation of Ovs within the nanotube, which facilitates CO adsorption; and (3) the anchoring of Au NPs, which captures electrons in the nanotube and promotes preferential O_2_ activation. Consequently, the optimal Au-CTOv shows 2.6-fold higher CO conversion rates compared to those of CTO nanotubes and is superior to N-doped TiO_2_. This work provides a unique strategy for designing and producing highly active titanate-based hollow photocatalytic materials.

## 2 Results and discussion

### 2.1 Design and synthetic pathway

**SCHEME 1:**
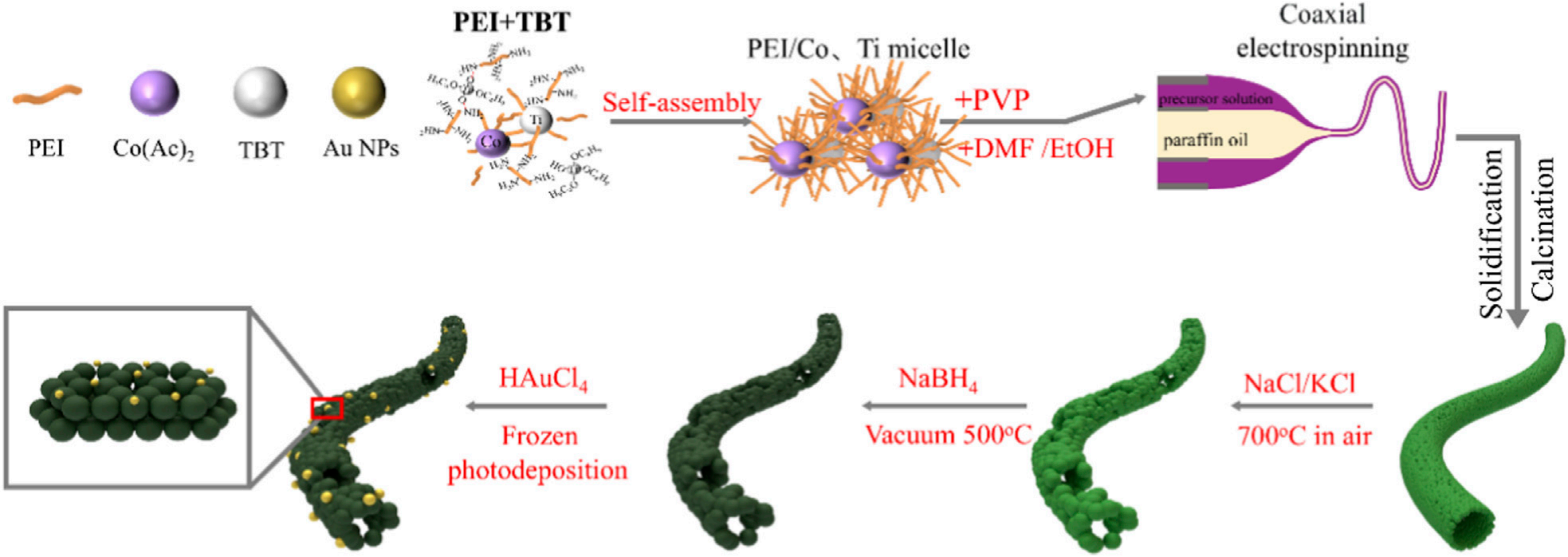
Schematic synthetic pathway and mechanism of Au-CoTiO_3_ nanotubes with oxygen vacancies (Au-CTOvs).

The designed and controlled synthesis process of Au-CTOvs is illustrated in Scheme 1. One-dimensional (1D) CoTiO_3_ nanotubes (CTO) are synthesized by coaxial electrospinning with Co(CH_3_COO)_2_ as the raw material, followed by the reaction with tetrabutyl titanate mixed with PEI to form CoTiO_3_ mesoporous precursors. Subsequent thermal treatment yields the CoTiO_3_ nanotubes. However, the formation of the pure rhombohedral phase of CoTiO_3_ requires heating to at least 650 °C, which causes the collapse of the pore structure, thereby degrading the photocatalytic performance. To address this issue, a molten salt method has been employed to fuse adjacent NPs, thereby increasing the interparticle gaps to form mass transfer voids and enhance the specific surface area. Two different eutectic molten salt systems, namely, LiCl/KCl and NaCl/KCl, are selected to modulate CTO due to their lower melting points and robust stability as they are known to yield CTO with favorable void structure and properties. Notably, the sample obtained from the LiCl/KCl eutectic molten flux consisted of other phases, i.e., Li_2_Ti_3_O_7_ and Co_2_TiO_4_, whereas pure ilmenite CoTiO_3_ (JCPDS 15–0866) crystals were grown from the NaCl/KCl eutectic flux (Supplementary Figure S1). This observation indicates that LiCl directly participates in the recrystallization of CoTiO_3_, rendering it unsuitable as a molten salt for this purpose. Subsequently, surface Ovs are engineered on the CTO by a vacuum-assisted hydrogen reduction strategy using NaBH_4_. The vacuum environment lowers the thermal decomposition temperature of NaBH_4_, generating a localized high H_2_ concentration gradient at the catalyst surface, enabling controlled Ov formation while minimizing bulk defects. Finally, a freezing photoreduction procedure is adopted to anchor Au NPs onto the Ov-modified CTO (denoted as Au-CTOvs), where the Au NPs are positioned around Ov sites in a negative charge accumulation environment.

### 2.2 Oxygen vacancy-created CoTiO_3_ nanotube

Initially, the samples are optimized by adjusting the calcination temperature, which is varied from 600 °C to 700 °C. The optimized CoTiO_3_ nanotubes are obtained by calcination at 650 °C, with an average tube diameter of 850 nm and a wall thickness of approximately 100 nm (Supplementary Figure S2). Lower annealing temperatures result in polycrystalline-phase nanotubes rather than the pure rhombohedral phase, owing to insufficient energy for stable atomic arrangement (Supplementary Figure S3a). In contrast, when the calcination temperature reaches 700 °C, the size of the CoTiO_3_ nanotubes increases significantly, leading to an obvious decrease in photocatalytic performance (Supplementary Figures S2c, S3c). Notably, the calcined CoTiO_3_ nanotubes become denser (Supplementary Figures S2a,b), which is due to the growth of CoTiO_3_ particles, resulting in the disappearance of the pore structure and consequently a significant reduction in the specific surface area during the high-temperature treatment (Supplementary Figure S3b). Considering earlier reports that the molten salt method can create large voids between particles during the particle recrystallization process (Wang et al., 2016), the changes in CoTiO_3_ before and after the molten salt treatment are investigated. The crystal phase and optical absorption of molten salt-treated CoTiO_3_ (CTO) are well inherited from the calcined CoTiO_3_ nanotube (Supplementary Figures S4a,b), but a noticeably looser structure and increased specific surface area of nanotubes are observed in the former, which contribute to improved photocatalytic performance (Supplementary Figures S4c–e).

To explore the influence of Ovs on nanotubes, nanotubes are optimized by adjusting the mass ratio between the CTO and NaBH_4_, which is represented as 4:1, 6:1, and 8:1. As depicted in Supplementary Figure S5a, as the mass ratio increases, minimal changes are discernible in both the crystallinity of the samples, and the creation of Ovs has no significant effect on void size. The enhancement in visible-light absorption is attributed to Ovs acting as impurity levels (Hoang et al., 2012), which expand the light absorption capacity of the nanotubes (Supplementary Figure S5b). Based on the results derived from photoluminescence (PL) and fluorescence (FS) spectra, which are related to the formed hydroxyl radical (·OH) amounts, the optimal mass ratio is 6:1 of NaBH_4_ to create Ovs beneficial for accelerating photogenerated charge separation (Supplementary Figures S5c,d). Consequently, the sample synthesized with a mass ratio of 6:1 exhibits the best photogenerated charge separation and photocatalytic CO oxidation performance (Supplementary Figure S5e) and is selected as the test sample, labeled as CTOvs, for subsequent experiments.

**FIGURE 1:**
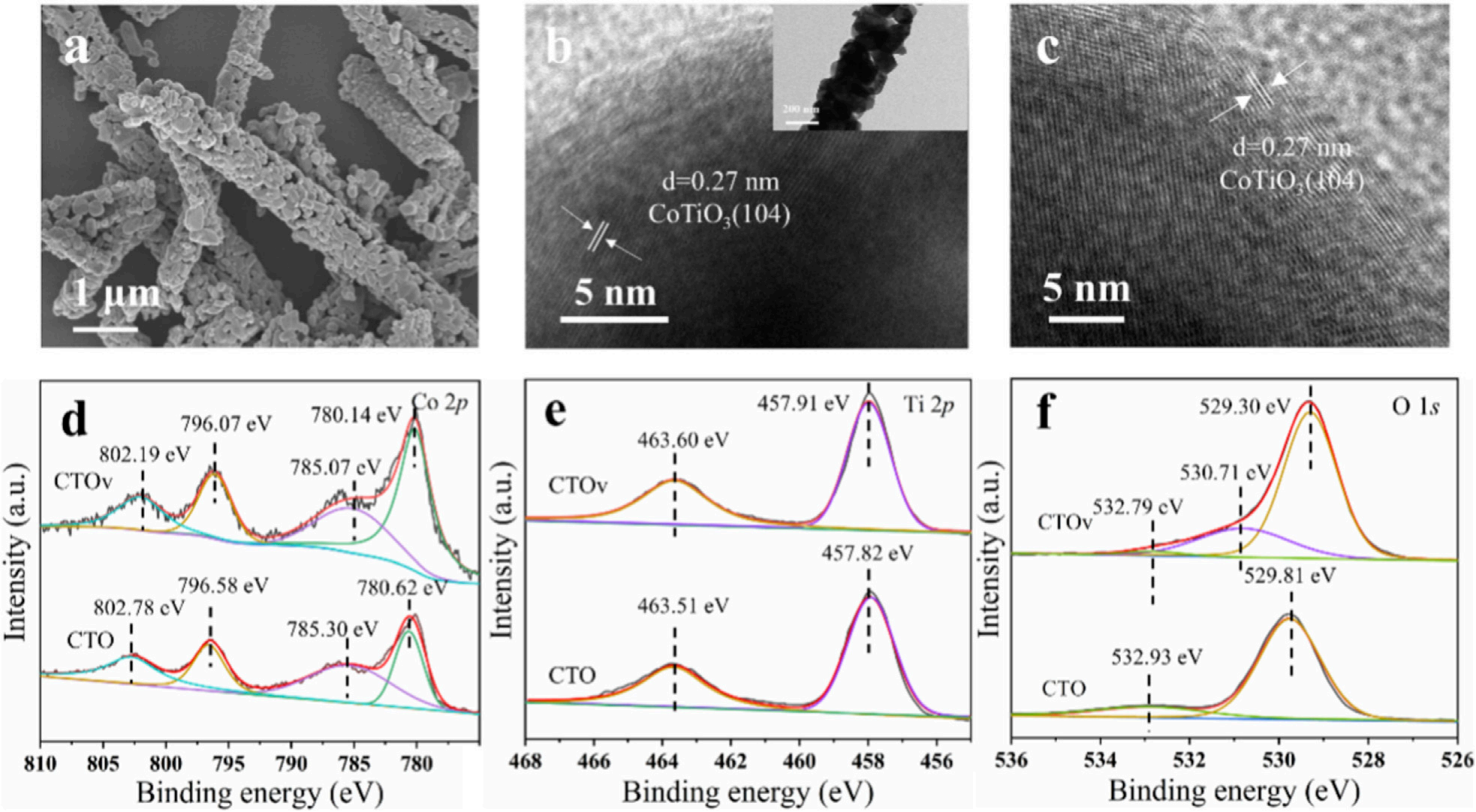
SEM images **(a)** and HRTEM image **(b)** (inset: TEM image) of CTOvs. HRTEM image of CTO **(c)**, Co 2*p* **(d)**, Ti 2*p* **(e)**, and O 1*s*. **(f)** XPS high-resolution spectra of CTO and CTOvs.

Furthermore, a comparative analysis of CTO and CTOvs is conducted, as shown in Figure 1a and Supplementary Figure S4c. The scanning electron microscopy (SEM) image shows that CTOvs has a noticeably loose tubular structure, with a diameter of approximately 850 nm and a wall thickness of approximately 100 nm, which are almost identical to those of CTO. The CTO and CTOvs are both composed of CoTiO_3_ NPs stacked along the tubes, and there are obviously large voids between the particles. Conspicuous lattice fringes with a d-spacing of 0.27 nm can be observed in the high-resolution transmission electron microscopy (HRTEM) image, corresponding to the (104) plane of ilmenite CoTiO_3_ (Figure 1b) (Su et al., 2022). However, the nanoparticle surface of CTOvs shows a disordered layer with a thickness of approximately 1 nm, which envelops the crystalline core. In contrast, high crystallinity can be observed throughout the CTO nanoparticles, showing well-resolved lattice fringes and crystal edges (Figure 1C). It might be due to the hydrogen reduction process introducing Ovs on the crystal surface, causing the crystalline structure to transform into a disordered one (Sun et al., 2017).

To further explore the role of the Ov structure in CTOvs and the chemical composition of the samples, X-ray photoelectron spectroscopy (XPS) analysis is performed on CTO and CTOvs. As shown in Supplementary Figure S6, the survey spectrum indicates the presence of Co, Ti, O, and C in both samples, without any trace of Na, K, or B, confirming that no impurities are introduced during the molten salt and hydrogen reduction process. Figures 1d–f show the high-resolution XPS spectra of Co 2*p*, Ti 2*p*, and O 1*s* of CTO and CTOvs, respectively. As shown in Figure 1c, for CTO, the peaks centered at 780.62 eV and 796.58 eV are assigned to the Co 2*p*_3/2_ and Co 2*p*_1/2_ states, respectively, suggesting the presence of Co^2+^ (Feng et al., 2022). The peaks with binding energies at 457.82 eV and 463.51 eV are characteristic of Ti 2*p*_3/2_ and Ti 2*p*_1/2_ states, respectively, matching well with the published data for Ti^4+^ of CoTiO_3_ (Figure 1d). Meanwhile, two O 1*s* peaks at 529.81 eV and 532.93 eV can be due to the crystal lattice oxygen in CoTiO_3_ and chemisorbed water molecules or surface hydroxyl groups, respectively (Figure 1e). It can be clearly observed that the area of the latter is significantly smaller than that of the former, which is attributed to the sample being subjected to multiple calcination steps, causing the hydroxyl groups adsorbed on its surfaces to desorb. Notably, a 0.5 eV shift toward lower binding energy occurs in the Co 2*p* spectra of CTOvs but not in that of CTO, indicating that the Co atoms are situated in a different chemical environment from those in CTO, likely due to the migration of holes toward Ovs (Papageorgiou et al., 2010). Meanwhile, combined with the result that no Ti^3+^ is identified, it can be concluded that the created Ovs on the CTO nanotube are shallow defects. In the O 1s spectra, a new peak at 531.0 eV is observed from the CTOvs, which is the signature of Ovs (Zhang Y. et al., 2025). Moreover, a similar shift is observed in the O 1*s* spectrum, where the binding energy at 529.81 eV is shifted to 529.30 eV, confirming that the Ov can serve as hole traps and promote the hole transfer (Kang et al., 2013). Based on this advantage, the CTOvs exhibits a higher photogenerated charge separation and photocatalytic activity for CO oxidation compared to the CTO (Supplementary Figure S5f).

### 2.3 Ultrafine Au NP-modified CTOvs

**FIGURE 2:**
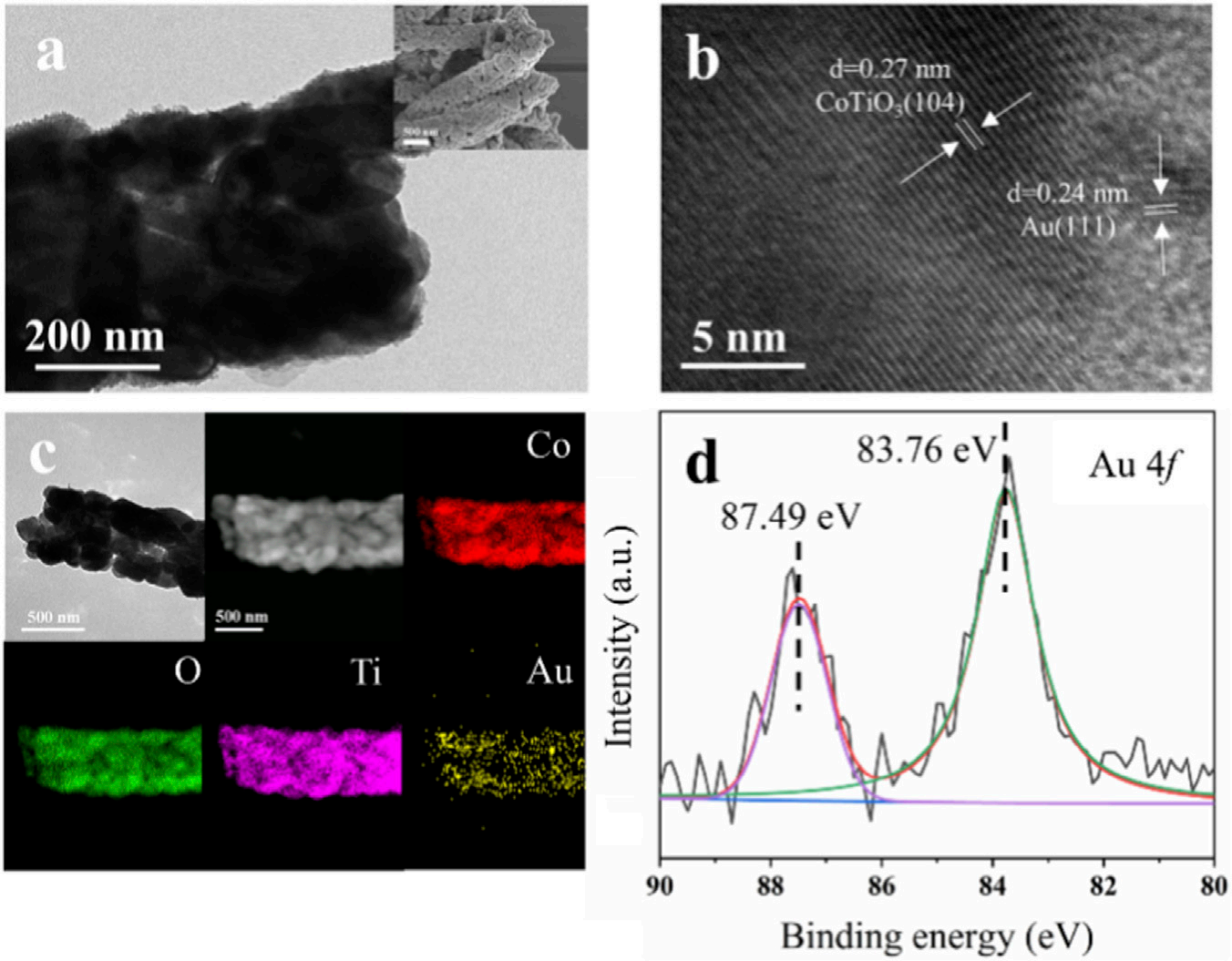
TEM image **(a)** (inset: SEM image), HRTEM image **(b)**, EDS elemental mapping images **(c)**, and Au 4*d* XPS high-resolution spectra **(d)** of the Au-CTOv. The areas highlighted in purple and yellow represent Au and CoTiO_3_ NPs, respectively.

The cocatalyst content significantly influences the catalytic performance; thus, CTOvs with different Au contents (xAu-CTOv, x = 0.5, 1, and 1.5) have been synthesized using a frozen photodeposition strategy. As illustrated in Figure 2a and Supplementary Figure S7a, the incorporation of Au NPs does not alter the spatial configuration or crystal structure of CTOvs. Furthermore, the HRTEM image (Figure 2b) of a selected area of Au-CTOvs confirms the deposition of Au NPs with a size below 2 nm onto the nanotubes, along with lattice fringes corresponding to the (111) plane of Au NPs. The energy-dispersive X-ray (EDX) elemental mapping images (Figure 2c) reveal that Au is relatively uniformly distributed on the nanotube surface, with localized regions of higher aggregation density. This may be attributed to the exposure of Ovs, leading to localized enrichment of photogenerated electrons during the deposition of Au. The crystal structure of all samples has been observed by XRD (Supplementary Figure S7a). It is to be expected that the introduction of Au NPs does not affect the crystal and texture structure of the CTOvs. Au NPs exhibit a pronounced plasmonic resonance absorption that intensifies with increasing Au content (Supplementary Figure S7b). The photocatalytic CO oxidation performance of the samples increases initially and then decreases with increasing Au content, peaking at 1% (Supplementary Figure S7c). As a result, the 1Au-CTOvs is selected as the test sample and labeled as Au-CTOvs.

To further demonstrate the effective combination of Au and CoTiO_3_, the CTOvs and Au-CTOvs are investigated by XPS measurements (Supplementary Figure S8a). The two peaks centered at 83.76 eV and 87.49 eV correspond to Au 4*f*_7/2_ and 4*f*_5/2_, respectively (Figure 2d) (Zhu et al., 2025). A slight positive shift has been noted in the high-resolution Co 2*p* and Ti 2*p* (Supplementary Figures S8b,c) binding energies of the Au-CTOvs compared to the CTOvs, which is attributed to electron transfer from CoTiO_3_ to Au at the Au/CoTiO_3_ interface, leading to a reduction in the outer electron cloud density of Co and Ti in the composite. Meanwhile, the area of Ovs in the Au-CTOvs is similar to that of the CTOvs (Supplementary Figure S8d), indicating that the incorporation of a small amount of Au has no effect on the Ovs on the nanotube surface. Combined with the TEM results, it is clarified that Au NPs display close interfacial contact with the surface of the CTOvs, achieved by the frozen photodeposition strategy.

**FIGURE 3:**
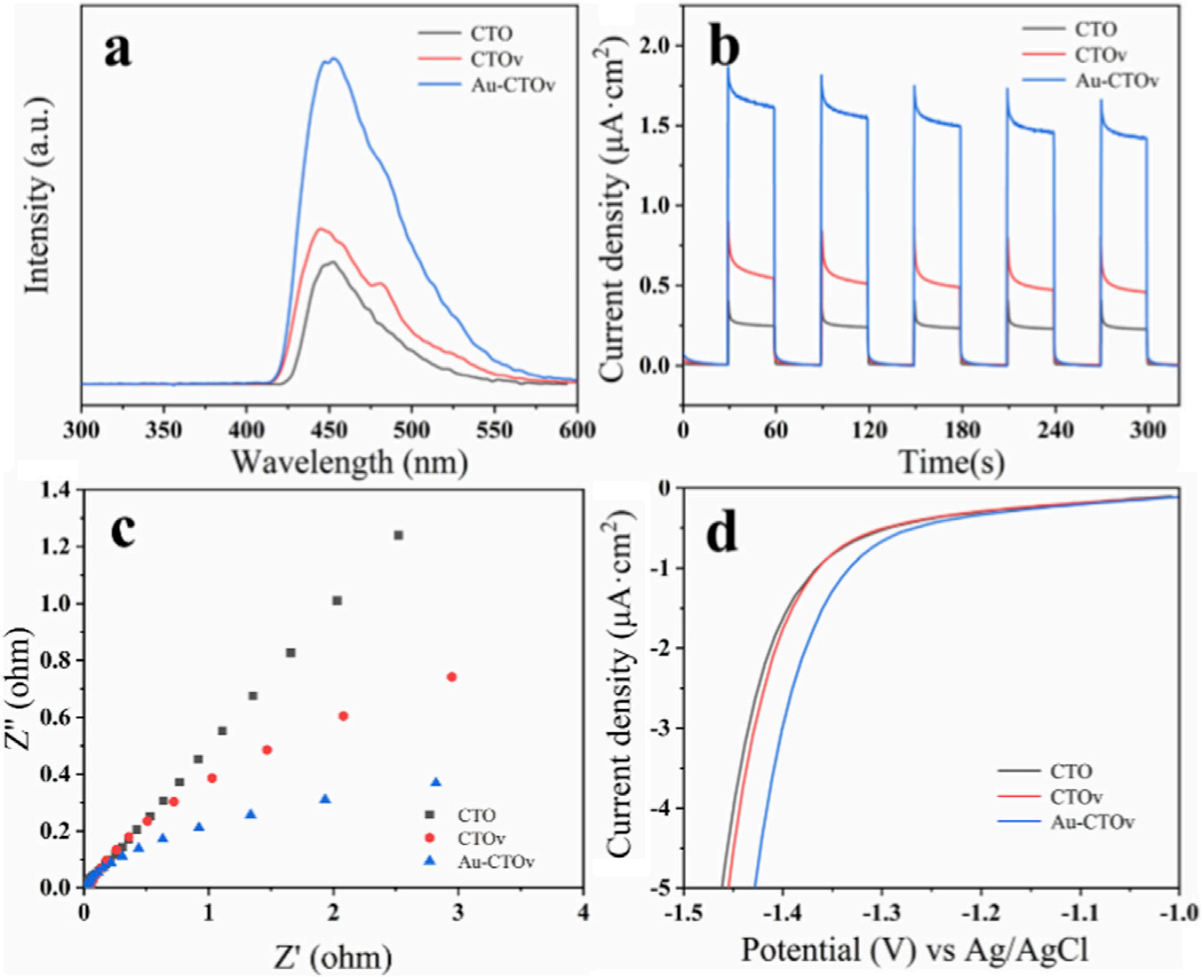
FS related to the amounts of **·**OH formed after irradiation for 0.5 h **(a)**, photocurrent response density measurement **(b)**, EIS curves **(c)**, and electrochemical reduction curves in O_2_-saturated electrolyte **(d)** of CTO, CTOvs, and Au-CTOvs.

The impact of Au incorporation on the photogenerated charge separation and O_2_ activation capacity of the CTOvs has been investigated. It is an effective method to investigate photogenerated charge separation by analyzing the intensity of the FS spectra related to the formed **·**OH radicals, the photocurrent response density, the electrochemical impedance spectra, and the electrochemical reduction curves. As displayed in Figure 3a, the FS signal intensity of Au-CTOvs is significantly heightened compared to that of CTOvs and CTO. This suggests an effective improvement in charge separation fostered by both the unique nanotube structure and the loaded Au NPs, benefiting the capture of photogenerated holes by H_2_O. Furthermore, the photocurrent response density is determined largely by the separation efficiency of the photogenerated electron–hole pairs within the photocatalyst. The higher photocurrent of Au-CTOvs indicates that the separation efficiency of photoinduced electrons and holes can be improved through the incorporation of Au and oxygen vacancy (Figure 3b). In the EIS test, the arc radius on the EIS spectra indicates the resistance of the material. A smaller semicircle radius reflects more effective separation of photogenerated electron–hole pairs and faster interfacial charge transfer to the electron donor or acceptor. Au-CTOvs display the smallest semicircle radius, indicating the lowest resistance among the samples and, consequently, the fastest interfacial charge carrier transfer (Figure 3c). Finally, electrochemical measurements are conducted to assess the catalytic function of Au NPs. Figure 3d displays the electrochemical reduction curves of the samples in an O_2_-saturated electrolyte, where Au-CTOvs show the lowest onset overpotential, suggesting that Au decoration improves O_2_ reduction. These results clearly demonstrate that the decorated Au NPs effectively capture electrons from CoTiO_3_ and facilitate O_2_ reduction.

**FIGURE 4:**
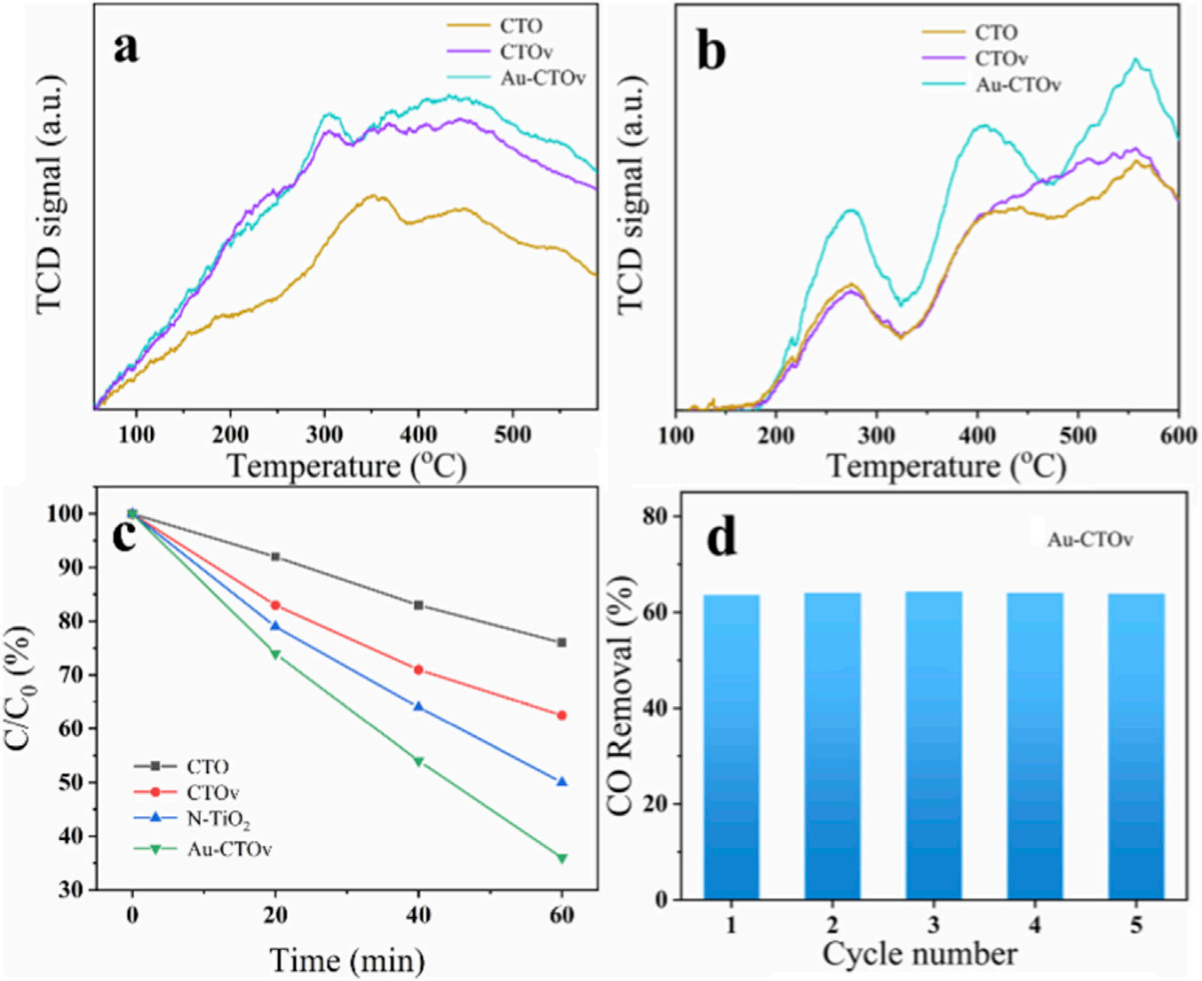
TPD curves for CO **(a)** and O_2_ **(b)** of CTO, CTOvs, and Au-CTOvs. Photoactivity for CO oxidation of CTO, CTOvs, commercial N-doped TiO_2_, and Au-CTOvs **(c)**. Photoactivity for CO oxidation of Au-CTOvs over five irradiation cycles **(d)**.

To explore the direct relationship between the CO and O_2_ adsorption capacities and the photocatalytic activity of the samples, CO temperature-programmed desorption (CO-TPD) and O_2_-TPD tests have been performed (Figures 4a,b). CTOvs shows significantly stronger CO adsorption than CTO, indicating that the creation of oxygen vacancies greatly improves the affinity of CO to CTOvs, which is consistent with the CO-TPD results. However, the CO adsorption on CTOvs is only slightly increased after Au modification, indicating that the Ovs mainly enhance CO adsorption. As illustrated in Figure 4b, CTOvs exhibits slightly higher O_2_ adsorption capacity than CTO due to the O_2_-attracting nature of oxygen vacancies. In comparison, the adsorption of O_2_ on CTOvs is greatly promoted after Au decoration. This indicates that Au NPs are more favorable for O_2_ adsorption, leading to robust O_2_ activation.

According to the above analysis, it is speculated that the Au-CTOvs has an exceptional photocatalytic CO oxidation performance. When matched against CTO and CTOvs, the CO oxidation conversion of the Au-CTOvs is significantly elevated, which is consistent with the result of charge separation analysis. The Au-CTOvs exhibits a major improvement in photocatalytic CO oxidation performance with enhancements approximately 1.3, 1.7, and 2.7 times greater than N-doped TiO_2_, CTOvs, and CTO, respectively (Figure 4c). All nanotubes possess voids and a hollow structure, with the nanotubes constructing a loose 3D network that facilitates mass diffusion for gaseous reactions. Meanwhile, the Ov in both Au-CTOvs and CTOvs improves CO adsorption to effectively capture photogenerated holes. Furthermore, the efficacy of Au NPs in promoting charge separation and providing more catalytic sites for O_2_ activation also contributed to the improvement in photoactivity. In addition, the photocatalytic activity of Au-CTOvs displays no obvious decline after five cycles (Figure 4d), indicating its good stability.

### 2.4 Discussion on mechanism

**FIGURE 5:**
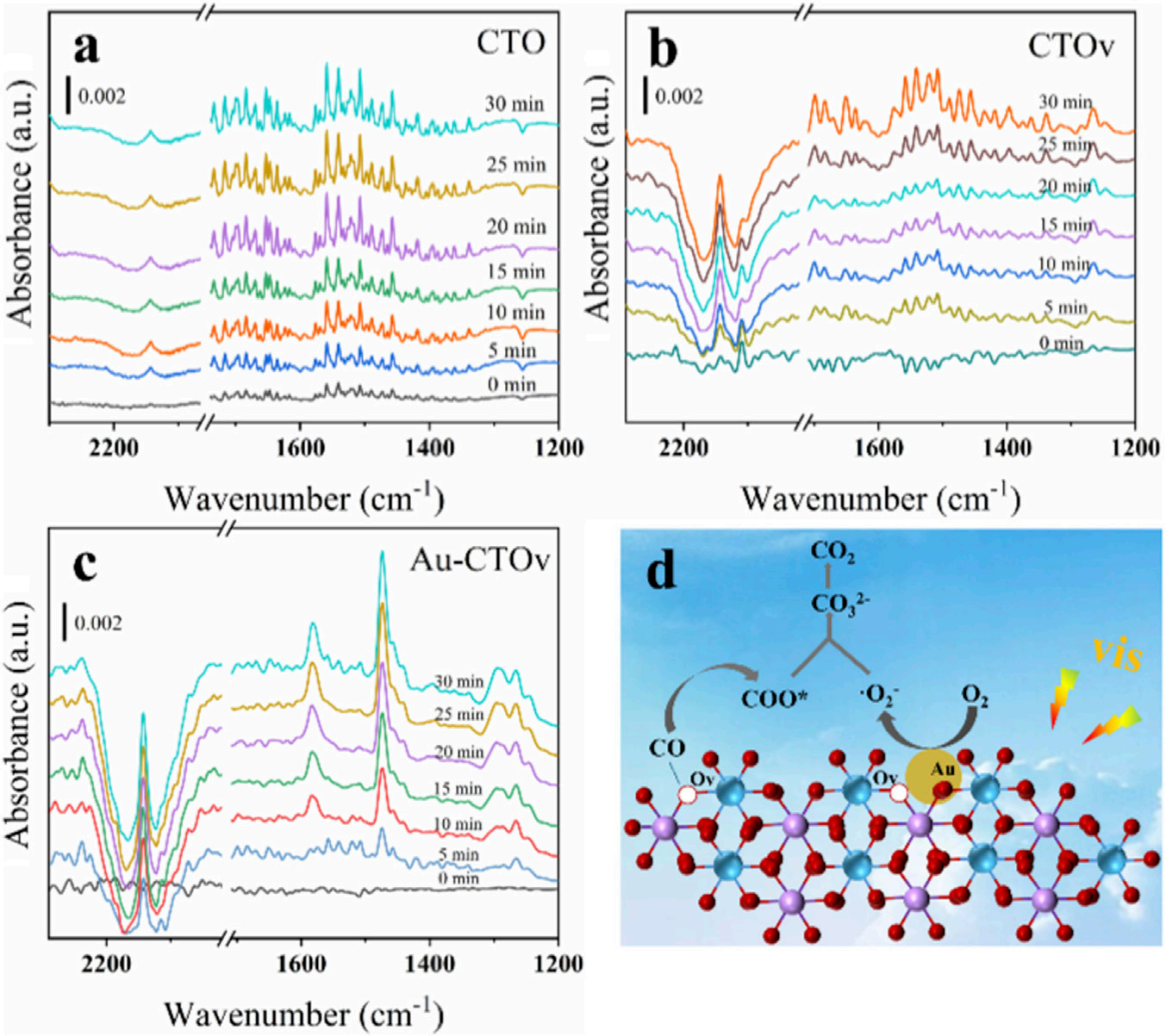
*In situ* FT-IR spectra with varying irradiation time on CTO **(a)**, CTOvs **(b)**, and Au-CTOvs **(c)**. Schematic illustration of photogenerated charge transfer and the CO oxidation process on Au-CTOvs **(d)**.

To elucidate the comprehensive photocatalytic reaction mechanism, particularly focusing on the catalytic oxidation process, we conducted *in situ* Fourier-transform infrared spectroscopy (FT-IR) to analyze reactant adsorption on representative samples. As shown in Figure 5, the infrared peaks at 2,117 and 2,173 cm^-1^ correspond to the stretching vibration of CO molecules adsorbed on the catalyst surface (Green et al., 2011). Simultaneously, under light illumination, a rapid formation of reaction intermediates is observed. The peaks at 1,600–1,400 cm^-1^ are likely attributed to the COO* intermediate, which forms when adsorbed CO on the Ov reacts with lattice oxygen (He B. et al., 2024). At this point, the initially physically adsorbed CO transforms into a chemically adsorbed one. Notably, the peaks at 1,575 and 1,477 cm^-1^ are assigned to carbonate (CO_3_^2-^) (Gaur et al., 2013). The species intensify gradually with an increasing irradiation time and are believed to originate from the conversion of COO* in the presence of reactive oxygen species (ROS). Finally, CO_3_^2-^ on the catalyst surface decomposes to produce CO_2_ and is released from the lattice oxygen (Chen et al. 2023). The conversion rate of intermediate species in the presence of ROS is the rate-limiting step of the CO oxidation process. The creation of Ovs into nanotubes attenuates the peaks of intermediate species (Figures 5a,b), suggesting that surface Ovs enhance charge separation, enabling the transfer of photogenerated electrons and facilitating the reduction of partial O_2_ to produce ROS. These ROS then react with intermediate species, promoting CO consumption. The incorporation of ultrafine Au NPs reduces the accumulation of intermediate species (Figure 5c), indicating that the reaction rate of these species is equal to or greater than their generation rate. This explains how Au promotes the conversion of intermediate species and accelerates the desorption of reaction products.

Based on the results and in conjunction with an *in situ* FT-IR analysis, a mechanism for possible charge separation and the associated CO oxidation reaction on Au-CTOvs is proposed (Figure 5d). The loosened hollow structure of CTO, fabricated through coaxial electrospinning and the molten salt method, enhances mass transfer efficiency. The created oxygen vacancies on CoTiO_3_ facilitate CO adsorption and effectively capture photogenerated holes. With the assistance of the lattice oxygen, they react with CO to form COO* intermediates. In parallel, the separated photoelectrons would be extracted by the modified Au and activate the adsorbed O_2_ to promote the production of RDS. The intermediate further reacts with ROS to generate CO_3_^2-^, which is deposited on the CoTiO_3_ surface. Consequently, the CO_3_^2-^ intermediate undergoes rapid decomposition, yielding CO_2_ and desorbing from the lattice oxygen, thereby freeing up catalytic sites for the subsequent oxidation reaction.

## 3 Conclusion

In summary, ultrafine Au NPs loaded on oxygen vacancy-CoTiO_3_ nanotubes (Au-CTOvs) have been successfully synthesized through a multi-step process, demonstrating excellent photocatalytic performance for CO oxidation. For the construction of these materials, CoTiO_3_ nanotubes are controllably fabricated using the coaxial electrospinning technique and molten salt method. Subsequently, Ovs are created within the CTO by a vacuum-assisted hydrogen reduction strategy using NaBH_4_. Finally, the *in situ* deposition of ultrafine Au NPs onto the surface of the nanotubes is controllably achieved through a frozen photodeposition strategy. The high photocatalytic activity of Au-CTOvs is primarily attributed to their void and hollow structures, which accelerate mass transfer and enhance O_2_ adsorption during the gaseous reaction. The creation of Ovs within nanotubes facilitates CO adsorption and the transfer of photogenerated holes. More importantly, the anchoring of ultrafine Au NPs on nanotubes serves as a catalytic site, capturing photogenerated electrons in nanotubes for efficient O_2_ activation. This study provides an essential strategy and reference for expanding the application of coaxial electrospinning technology in constructing heterojunction photocatalytic materials.

## Data Availability

The original contributions presented in the study are included in the article/Supplementary Material; further inquiries can be directed to the corresponding authors.
