# Supplementary material for: Improved visible photocatalytic CO oxidation of CoTiO3 nanotubes by surface engineering and Au nanoparticle loading
Source: Front Chem. 2026 Aug 3;14:1884025. doi: 10.3389/fchem.2026.1884025 (PMC13479130; doi:10.3389/fchem.2026.1884025)
Supplement: Supplementary file 1 [file DataSheet1.docx]

Supplementary Material

Improved visible photocatalytic CO oxidation of CoTiO_3_ nanotube by surface engineering and Au nanoparticles loading

Zhanyu Chu, TianWei Dou, Zhuo Li, Lei Sun*, Liqiang Jing*

Key Laboratory of Functional Inorganic Materials Chemistry (Ministry of Education), School of Chemistry and Materials Science, International Joint Research Center for Catalytic Technology, Heilongjiang University, Harbin 150080, P. R. China.

*Corresponding author, e-mail address: 2021022@hlju.edu.cn (L. Sun), jinglq@hlju.edu.cn (L. Jing)

# 1 Experimental Section

## 1.1 Materials Synthesis

1.1.1 **Reagents and Solvents**

Cobalt acetate tetrahydrate (Co(CH_3_COO)_2_, 99%, Aladdin), tetrabutyl titanate (TBT, 99%, Aladdin), polyethyleneimine (PEI, Mw~600, 99%, Aladdin), polyvinylpyrrolidone (PVP, Mw~1300000, Aladdin), hydrochloric acid (HCl, 37%, Tianjin Fuyu Fine Chemical Co. Ltd.), N,N-dimethylformamide (DMF, 99.5%, Tianjin Fuyu Fine Chemical Co. Ltd.), ethanol (99.5%, Tianjin Fuyu Fine Chemical Co. Ltd.), paraffin oil (Tianjin Fuyu Fine Chemical Co. Ltd.) and chloroauric acid (HAuCl_4_, 35%, Sigma-Aldrich.) were all used as-received without further purification. Deionized water (18.2 MΩ) was purified by a Millipore purification system.

1.1.2 **Synthesis of the CoTiO_3_ nanotube**

The CoTiO_3_ nanotube were fabricated via coaxial electrospinning following this optimized protocol. Typically, 3 g TBT and 0.4 g Co(CH_3_COO)_2_ were magnetically stirred for 1 h as solution A. A mixed solution containing 1 g DMF and 3 g ethanol as solution B. Then, the spinning solution was formulated by homogenizing solutions A and B with 0.3 g PVP under continuous stirring for 12 h. Subsequently, the spinning solution and paraffin oil were transferred into the coaxial electrospinning apparatus as shell and core solution, respectively. The nanotubes were prepared using a coaxial electrospinning system (working distance of 20 cm, the voltage of 15 kV, 18# steel needle as the outer and 25# steel needle as the inner, outer and inner injection rate at 0.8 and 0.2 mL h^-1^, respectively, temperature about 30^o^C and humidity below 30%). The nanotubes were dried at 80 ^o^C for 12 h, and calcined at 650^o^C (1^o^C min^-1^ ramp) for 2 h in air to obtain as-synthestized CoTiO_3_ nanotube.

1.1.3 **Synthesis of CTO**

Mixed different types of eutectic molten salts uniformly, and grind them into a powder using a mortar. Then added 100 mg of CoTiO_3_ into the mortar and gently grind to mix evenly. Transfer the sample into a crucible and place it in a muffle furnace. Heat the sample to different temperatures (650 °C, 700 °C and 750 °C) at a constant heating rate of 5 °C min^-1^. After the crucible cool to room temperature, added deionized water to soak for 1 h, then performed suction filtration to remove the mixed salts. Washed the sample thoroughly, transfer it to a drying oven at 60 °C for 12 h, yielding one-dimensional porous CoTiO_3_ nanotubes, labeled as CTO.

1.1.4 **Synthesis of CTOv**

In a typical synthesis, CTO (100 mg) and NaBH_4_ (25 mg, 16.67 mg, and 12.5 mg, which corresponding to mass ratios of 1:4, 1:6, and 1:8, respectively) were separately placed in two rectangular porcelain crucibles in the vacuum tube furnace. Then, CTO was held on top of the NaBH_4_ and rotated by a certain angle relative to each other. The furnace was sealed, subjected to vacuum treatment (pressure < 0.001 MPa), and then heated to 500 ^o^C to generate a high concentration of H_2_ in a localized area by the thermal decomposition of NaBH_4_. The product was obtained after 2 h of treatment with a heating rate of 5 ^o^C min^-1^, which is denoted as CTOv.

1.1.5 **Synthesis of Au-CTOv**

Au-CTOv were synthesized via a frozen photodeposition strategy. Typically, 50 mg of pre-synthesized CTOv were uniformly dispersed in deionized water under magnetic stirring for 30 min. A certain amount of HAuCl_4_ were then introduced into the suspension, followed by continuous stirring in the dark for 30 min. The mixture was subsequently flash-frozen in liquid nitrogen and irradiated under UV light for 10 min to facilitate Au deposition. After thawing, the product was centrifuged, sequentially washed with ethanol and deionized water to remove impurities, and finally dried at 80 ^o^C overnight. The resulting samples were labeled as xAu-CTOv, where x (0.5, 1 and 1.5) denotes the mass percentage of Au relative to CTOv.

## 1.2 Characterization of Materials

X-ray diffraction (XRD) was used with Cu kα radiation to record patterns on a Bruker D8 advance. N_2_ adsorption-desorption tests were determined by Quanta Chrome Autosorb iQ. Scanning electron microscopy (SEM) uses a ZEISS-sigma 500 instrument at a 5 kV voltage. Transmission electron microscopy (TEM) images were taken using a JEOLJEM-2010 electron microscope at a 200 kV voltage. X-ray photoelectron spectra (XPS) were recorded on a Kratos-AXIS ULTRA DLD instrument. UV–vis diffuse absorption spectra curves were measured using a Shimadzu UV-2700 spectrophotometer with BaSO_4_ as the basic carrier. Fourier transform infrared spectroscopy (FT-IR) characterization tests were conducted using a Thermo Scientific Nicolet iS50 with KBr as a diluent. Surface photovoltage spectroscopy (SPS) was surveyed with a self-built apparatus under oxygen atmospheres, equipped with a lock-in amplifier (SR830, USA) synchronized with a light chopper (SR540, USA). The light intensity employed in the cryogenic photodeposition experiment was 100 mW cm^-2^.

## 1.3 Hydroxyl Radical Production Analysis

20 mg photocatalyst was dispersed in 50 mL coumarin aqueous solution (1 mM). The adsorption-desorption equilibrium was achieved by vortex mixing stirred in dark for 10 min. After irradiated under 150 W Xe lamp (GYZ220) for 30 min, an appropriate amount of solution was centrifuged and transferred to a Pyrex cuvette for fluorescence measurement of 7-hydroxycoumarin at a 332 nm excitation wavelength through a spectrofluorometer (Perkin-Elmer LS55).

## 1.4 Photoelectrochemical Test

Photoelectrochemical experiments were performed using a typical three-electrode system in a glass cell with a 0.5 M Na_2_SO_4_ solution (pH = 6.8). The working electrode was prepared by a thin film electrode based on FTO via the scraping method. Pt wire served as the counter electrode and Ag/AgCl was the reference electrode. Photoelectrochemical performance was tested using an IVIUM V13806 electrochemical workstation and 300 W Xe lamp with a UV cutoff filter (>400 nm) as the illumination source. The temperature was kept at ambient temperature for all experiments. The photocurrent response density measurement was recorded with the bias voltage of 0.4 V.

## 1.5 *In Situ* Fourier Transform Infrared Spectra

*In-situ* Fourier transform infrared spectra (*in-situ* FTIR) were performed with an FTIR spectrometer of Nicolet IS50 to record the working curve. The experimental settings include using an MCT detector with a resolution of 4 cm^-1^ and scanning 16 for the samples, with an examination range of 1000 cm^-1^ to 4000 cm^-1^. The samples mixed with KBr were compressed and held in a custom-fabricated IR reaction chamber sealed with ZnSe windows. The catalyst was purged with N_2_ (flow rate at 40 mL min^-1^) at 170^o^C for 2 h to remove impurity gas adsorbed on the surface before measurement. After cooled to 25^o^C, the spectrum of the sample was collected as a collection background. Then, high purity O_2_ was introduced into the reaction chamber (flow rate at 40 mL min^-1^ for 30 min). Next, 5 vol% CO balanced with high purity O_2_ as the probe gas was introduced into the system with a total flow rate of 40 mL min^-1^ for 1 min and balanced for 30 min in a sealed reaction chamber, with continuous data collection.

## 1.6 Evaluation of Photocatalytic Activity

The photoactivities of the samples were investigated by measuring the photocatalytic oxidation rates of CO. To evaluate the recyclability and stability of the photocatalyst, after each cycle, the catalyst was washed with deionized water and ethanol in turn, then dried at 80 ^o^C to evaporate all absorbed reactants. The reaction was carried out in a reactor of about 600 mL equipped with thermostatic water. After filled the reaction vessel with O_2_ and injected 0.6 mL of CO into it, the CO concentration in the reactor was 1000 ppm. The CO concentration was detected using a gas chromatograph of GC2002 with a methane conversion furnace and FID detector. After the gas was diffused evenly for 30 min, 0.5 mL of gas was extracted from the reactor and injected into the gas chromatography to measure the peak area of CO (S0). The peak area of the remaining CO (St) in the reactor was measured after irradiation for 1 h with 300 W Xe lamp, and the light intensity was estimated to be 400 mW cm^-2^. Within a certain concentration range, the peak area of CO was directly proportional to the actual content, allowing for the calculation of the photocatalytic CO oxidation activities of the resulting samples. For comparison, blank experiments without O_2_, light or catalyst were also carried out, respectively.

# 2 Supplementary Figures

**Supplementary Figure 1.** XRD patterns of CTO obtains from the LiCl/KCl and NaCl/KCl eutectic molten flux by molten salt method, respectively.

**Supplementary Figure 2.** The SEM image of CoTiO_3_ nanotube obtains from different calcined temperature. 600 ^o^C (a), 650 ^o^C (b), 700 ^o^C (c).

**Supplementary Figure 3.** XRD patterns (a), N_2_ adsorption-desorption isotherm curves (b) and photoactivity for CO oxidation (c) of CoTiO_3_ nanotube obtains from different calcined temperature.

**Supplementary Figure 4.** XRD patterns (a), UV-vis absorption spectra (b), SEM image (c), N_2_ adsorption-desorption isotherm curves (d) and photoactivity for CO oxidation (e) of calcined CoTiO_3_ and molten salt-treated CoTiO_3_ nanotube, the HRTEM image (f) of molten salt-treated CoTiO_3_ nanotube.

**Supplementary Figure 5.** XRD patterns (a), UV-vis absorption spectra (b), PL (c), FS related to the amounts of ·OH formed after irradiation for 0.5 h (d) and photoactivity for CO oxidation (e) of CTO and CTOv obtains from different mass ratio between the CTO and NaBH_4_.

**Supplementary Figure 6.** XPS spectra CTO and CTOv.

**Supplementary Figure 7.** XRD patterns (a), UV-vis absorption spectra (b) and photoactivity for CO oxidation (c) of different xAu-CTOv.

**Supplementary Figure 8.** XPS spectra (a), Co 2*p* (b), Ti 2*p* (c) and O 1*s* (d) XPS high-resolution spectra of CTOv and Au-CTOv.

**Supplementary Figure 9.** XRD patterns (a) and UV-vis absorption spectra (b) of before reaction and after reaction of xAu-CTOv.
